# Impact of 1-h memory tasks with different cognitive loads on ET-derived indices of neurotransmitter-system involvement in university athletes

**DOI:** 10.3389/fpsyg.2026.1710518

**Published:** 2026-02-16

**Authors:** Xiaoyu Zhang, Ling Lin, Huaye Wang, Meixian Dai

**Affiliations:** Department of Physical Education, Ningbo University, Ningbo, China

**Keywords:** central fatigue, cognitive/mental fatigue, encephalofluctuogram technology (ET), ET-derived S-spectrum indices, memory task

## Abstract

**Purpose:**

To investigate the effects of memory tasks of different difficulty levels on the brain functional state of collegiate athletes and to explore the underlying neurophysiological mechanisms related to cognitive fatigue.

**Methods:**

Forty-five collegiate athletes (national second-level or above) were randomly assigned to a moderate-difficulty task group, a low-difficulty task group, or a control group (*n* = 15 each). A three-group pre–post design was used, with ET recorded at baseline and immediately after a 1-h intervention period. Accuracy and reaction time were continuously recorded. Neurophysiological outcomes were ET-derived S-spectrum indices (S1, S2, S4, S5, S6, S7, S11, S13) obtained using Encephalofluctuogram Technology (ET). Following prior ET reports, these indices were treated as indirect, model-derived proxy measures of neurotransmitter-system involvement rather than direct neurotransmitter measurements. Data were analyzed with repeated-measures ANOVA with Group (3) × Time (2).

**Results:**

The moderate-difficulty task led to a significant decline in accuracy and a block-wise slow–fast pattern in reaction time (*p* < 0.05), whereas no significant behavioral changes were found in the low-difficulty group. The ET-derived S4 index (previously proposed to reflect serotonergic-system involvement in ET studies) showed significant within-group, between-group, and interaction effects (all *p* < 0.01). No other ET indices showed robust changes after multiplicity correction.

**Conclusion:**

A 1-h moderate-difficulty memory task caused a progressive decline in accuracy with reaction times initially slowing and then accelerating, and was accompanied by an increase in the ET-derived S4 index alongside a smaller non-specific time effect in S11. These ET findings should be interpreted as indirect indices and are hypothesis-generating with respect to neurotransmitter mechanisms.

## Introduction

Long-term exposure to high-intensity training and competitive environments with elevated psychological stress often leads athletes to experience various forms of physiological and psychological fatigue, including fatigue states that can include both (i) exercise-related central fatigue discussed in sport physiology and (ii) mental/cognitive fatigue discussed in neuropsychology (Marcora et al., 2009; Van Cutsem et al., 2017). Such fatigue can subsequently impair athletes' performance and well-being in daily life, training, and competition. Importantly, mental/cognitive fatigue is also prevalent in non-athlete populations (e.g., students, office workers, and some clinical groups), making laboratory paradigms and mechanistic interpretation relevant beyond sport settings. With the rapid advancement of neurophysiological techniques, exercise-induced central fatigue has emerged as one of the major research focuses in the field of sport science.

Exercise-induced central fatigue generally refers to the phenomenon of mental fatigue resulting from excessive exercise-related cognitive and neural load, which leads to functional decline or dysregulation of the central nervous system (CNS). In sport physiology, “central fatigue” is often defined more broadly as a reduction in the CNS's ability to drive the motor system (distinct from peripheral fatigue), whereas neuropsychology typically uses “mental/cognitive fatigue” to describe reduced alertness/vigilance, impaired executive control, and performance decrements after prolonged cognitive demands. In the present paper, we focus on cognitive-task–induced fatigue in athletes, and we use the term “central fatigue” in the sport-science sense while explicitly aligning it with the neuropsychological construct of mental/cognitive fatigue (Gandevia, 2001; Meeusen et al., 2006; Meeusen and Roelands, 2018). Based on previous animal experiments, researchers in the field of sports science have reached a general consensus regarding the neurophysiological mechanisms underlying exercise-induced central fatigue (Chaouloff et al., 1989; Blomstrand et al., 1995; Dwyer and Flynn, 2002; Xu et al., 2008; Liu et al., 2017). Specifically, central fatigue induced by high-intensity training is associated with a reduction in excitatory neurotransmitters such as dopamine (DA), acetylcholine (Ach), and glutamate (Glu), along with an increase in inhibitory neurotransmitters including serotonin (5-hydroxytryptamine, 5-HT) and gamma-aminobutyric acid (GABA; Nie and Huang, 2003; Meeusen et al., 2006). However, research on whether cognitive tasks can induce central fatigue in athletes and the associated neurotransmitter mechanisms remains limited.

To better connect with neuropsychology, cognitive-task–induced fatigue can be situated within established frameworks of attention/alerting, working memory, and executive control. For example, sustained performance demands can lead to vigilance decrements (reduced alertness) and altered top-down control, and fatigue effects are often discussed in relation to executive functions such as updating, inhibition, and shifting, as well as dual-task costs (Warm et al., 2008; Miyake et al., 2000; Boksem et al., 2005). Current laboratory paradigms and techniques for central fatigue research are mainly based on the effects of prolonged cognitive tasks on neurophysiological indicators of the brain. These techniques include functional magnetic resonance imaging (fMRI), event-related potentials (ERP), electroencephalography (EEG), and eye-tracking systems. Due to the limitations inherent to these methods, they can only reveal how cognitive tasks affect electrophysiological signals related to cognitive functions and changes in cerebral blood flow and oxygen distribution. They do not directly quantify neurotransmitter concentrations in the way that neurochemical gold-standard approaches (e.g., PET ligands, microdialysis, CSF assays) aim to do; thus, studies often rely on *indirect* neurophysiological proxies when discussing neurotransmitter involvement (Roelands et al., 2021).

In the 1990s, a novel technique termed Encephalofluctuogram Technology (ET) was first developed in the field of aerospace research in China. ET provides a valuable tool for exploring neurotransmitter mechanisms underlying central fatigue and has opened a new avenue for in-depth investigations. As an original Chinese innovation, ET is a non-invasive technique that enables real-time monitoring of brain function. Technically, ET analyzes EEG-derived ultra-slow spectral fluctuations (S-spectrum; mHz range) obtained through power-spectrum fluctuation analysis, and outputs a set of spectral indices (S1, S2, …) describing these ultra-slow components (Zhou et al., 2009; Mei, 1994). In prior ET clinical literature, specific S-components have been assigned/used as indirect indicators of neurotransmitter-system involvement (e.g., S1–GABA, S2–Glu, S4–5-HT, S5–ACh, S7–NE, S11–DA), and group differences in these indices have been reported in patient samples (Zeng et al., 2005). Importantly, these are indirect, model-based indices rather than direct neurotransmitter measurements, and we therefore use cautious phrasing throughout (e.g., “ET-derived S4 index, interpreted as serotonergic involvement”). ET has been successfully applied in the sports domain, including athlete selection, performance evaluation, central adaptation to training, and detection of central fatigue (Lin and Liu, 2014; Liu et al., 2016). For example, domestic studies have found that ET can be used to assess central fatigue in athletes, and the ET fatigue index is correlated with heart rate variability (HRV), behavioral indicators, and subjective ratings of perceived exertion (RPE; Liu et al., 2016). Other studies have also suggested that changes in ET spectral features, such as the S-characteristic spectrum, can serve as markers for evaluating central fatigue in athletes (Lin and Liu, 2014; Meeusen and Roelands, 2018; Zhang et al., 2021). For international neuropsychology readers, it is also necessary to clarify the current validation status and limitations of ET. Available reports describe ET as a non-invasive indirect approach and cite comparisons where ET-tracked patterns were similar to CSF HPLC trends for Glu across post-stroke progression, as well as consistency between ET and plasma 5-HT in evaluating therapeutic effects in post-stroke depression; however, such evidence remains limited and does not equate to broad validation against gold standards across contexts (Yao et al., 2018). Accordingly, ET results should be interpreted as proxy indices with constraints in spatial specificity and inferences about neurotransmitter mechanisms.

Memory ability is of great significance for athletes, as their training, competition, daily life, and learning all involve a substantial number of memory-related tasks (Lebeck, 2024; El-Gazzar, 2025). As one of the mainstream paradigms of cognitive operations, memory tasks have been widely applied across various fields (Challis et al., 1996; Salmon et al., 1996; Cocchini et al., 2002). Critically, the cognitive-load manipulation in memory tasks can be theoretically linked to sustained attention/alertness demands and executive control (updating/inhibition), which are core components in neuropsychological models of mental fatigue and dual-task performance. However, to date, no studies have investigated the relationship between memory-task-induced central fatigue and neurotransmitter mechanisms.

Against this background, the present study aims to address the following key questions: Can memory tasks with relatively high cognitive loads induce central fatigue (central fatigue in the sport-science sense) in athletes? How do such tasks affect ET-derived S-spectrum indices that have been proposed in the ET literature to reflect neurotransmitter-system involvement (indirect proxies, not direct measurements)? Do tasks with different cognitive loads produce distinct patterns in behavioral fatigue markers, subjective fatigue, and ET-derived indices? To address these questions, and drawing on previous studies, preliminary experiments, and the specific aims of this research, the present study employed 1-h memory tasks of different difficulty levels as experimental manipulations. Behavioral performance and ET-derived indices were measured before and after task execution across groups. The purpose was to examine whether 1-h memory tasks of varying difficulty can induce cognitive/mental fatigue and trigger changes in ET-derived S-spectrum components that are interpreted as proxy indices of neurotransmitter-system involvement. Given the pilot/exploratory design, mechanistic interpretations are framed as hypothesis-generating rather than confirmatory. We hypothesized that: (1) 1-h memory tasks of moderate difficulty would reduce accuracy and alter reaction time in university athletes; and (2) moderate and low difficulty tasks would differentially affect ET-derived indices (e.g., S-components previously associated with inhibitory/excitatory/modulatory systems) rather than directly measured neurotransmitter concentrations.

## Materials and methods

### Participants

A total of 45 student athletes from the School of Physical Education at Ningbo University (all certified as National Level II athletes or above) participated in the study. Their mean age was 22.87 years (SD = 1.81). To characterize sample homogeneity, we recorded demographic and training variables; participants' primary sports were ball games (mainly soccer and basketball). Participants reported no history of neurological or psychiatric disorders and no current use of psychoactive medication. Participants were randomly assigned to one of three groups: Experimental Group 1, consisting of 15 individuals (9 males, 6 females) who performed a memory task of moderate difficulty. Experimental Group 2, consisting of 15 individuals (10 males, 5 females) who performed a memory task of low difficulty. And the Control Group, consisting of 15 individuals (8 males, 7 females) who did not engage in any cognitive task and were instructed to either rest quietly or engage in light free activities while remaining awake in the laboratory. To reduce between-participant variability, randomization was stratified by sex (male/female) and sport type (different ball sports), using a computer-generated allocation list prepared by a researcher not involved in testing. Allocation was concealed in sequentially numbered, opaque, sealed envelopes opened only after baseline assessments were completed. The inclusion criteria for participants were as follows: (1) Normal visual acuity, either unaided or corrected. (2) Right-handedness. All participants were right-handed and used the mouse with their right hand. (3) Good physical and mental health, with no recent use of medication. (4) No engagement in strenuous or exhaustive physical activity on the day prior to the experiment. (5) No alcohol consumption or overeating on the day prior to the experiment. (6) Adequate rest before the experiment, free from academic examinations or other stressors, and with a stable mental state. (7) Prior to the experiment, participants completed the Athlete Burnout Questionnaire (ABQ) and ET tests. According to the normative standards provided by Beijing Tongren Optoelectronics Company, participants showing signs of psychological fatigue (ABQ; Raedeke and Smith, 2001) or central fatigue (ET) were excluded to ensure that only non-fatigued participants took part in the study. Compliance with pre-test instructions (sleep, caffeine/alcohol, and training restriction) was checked using a brief self-report checklist upon arrival; participants who reported non-compliance were rescheduled or excluded (see recruitment flow below). Recruitment and exclusions were documented as follows: initially assessed for eligibility (*n* = 53); excluded prior to randomization (*n* = 8) due to (i) ABQ indicating burnout/fatigue (*n* = 3), (ii) ET indicating fatigue (*n* = 3), (iii) non-compliance with pre-test instructions (*n* = 2). All randomized participants completed the protocol and were included in the final analyses (per group *n* = 15), unless otherwise stated. This study was conducted in accordance with the Declaration of Helsinki (6th revision). All participants were informed of the experimental procedures and provided written consent prior to participation.

### Experimental design

**Figure 1:**
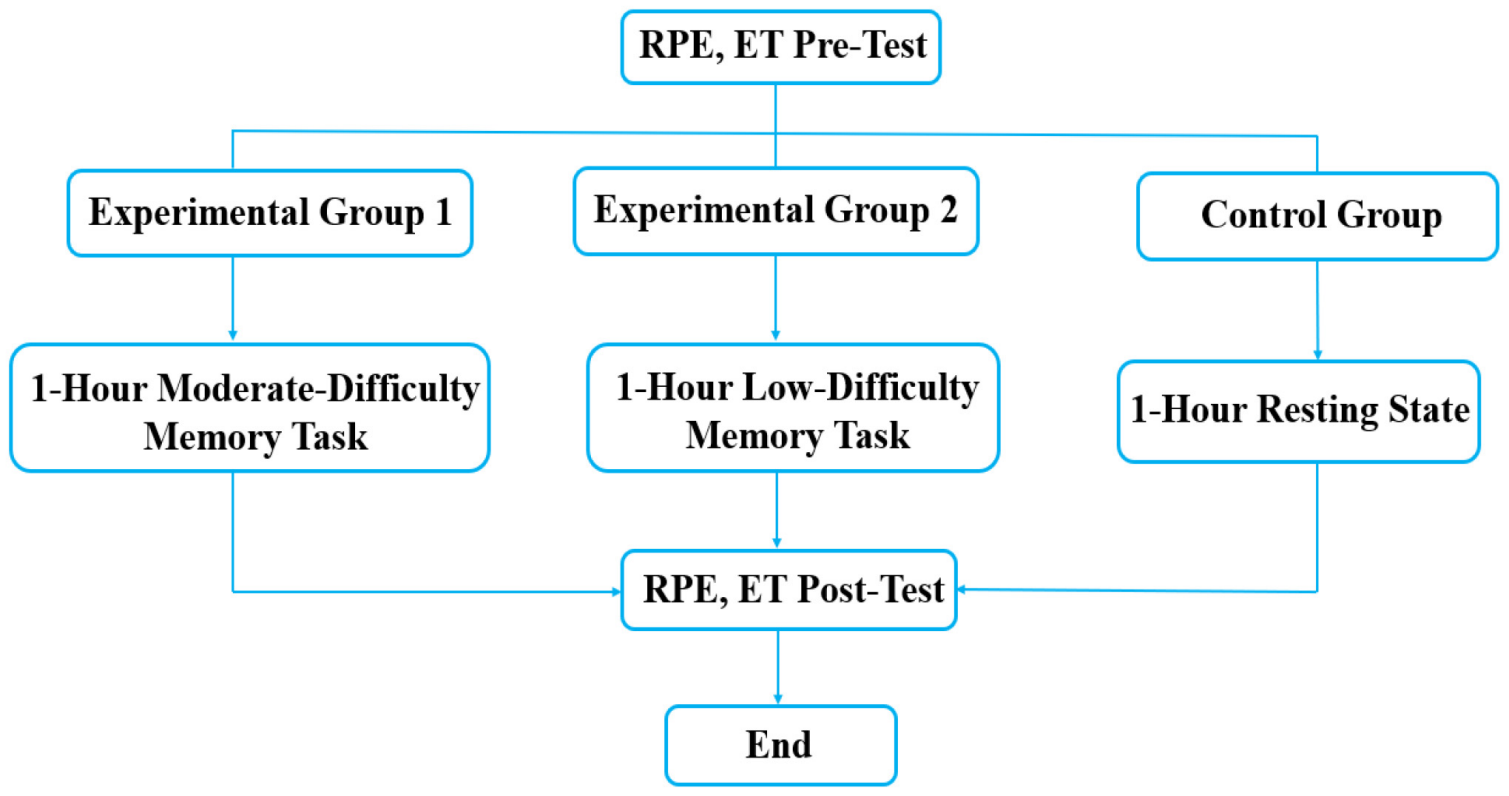
Flowchart of the experimental procedure. RPE, subjective fatigue questionnaire test; ET, encephalofluctuogram technology test.

This study employed a 3 (moderate-difficulty memory task, low-difficulty memory task, no cognitive task) × 2 (pre-test, post-test) experimental design. The between-subjects factor was the type of 1-h memory task with three levels, and the within-subjects factor was time. Dependent variables included behavioral measures (accuracy and reaction time) and ET-derived neurophysiological indices (S1, S2, S4, S5, S6, S7, S11, S13) that have been proposed in prior ET literature to relate to neurotransmitter-system involvement (e.g., GABAergic, glutamatergic, serotonergic, dopaminergic).Neurophysiological outcomes were ET-derived indices that are interpreted as reflecting involvement of neurotransmitter systems, rather than direct neurotransmitter concentrations. To assess subjective fatigue, the RPE was administered before and after the 1-h intervention. RPE was assessed using the Borg CR10 scale with standardized anchors explained to each participant before baseline assessment; participants pointed to a number on a printed scale, and the assessor recorded the score. The detailed experimental procedure is shown in Figure 1. Given the pilot/exploratory nature of this study, the sample size was determined by feasibility rather than an a priori power analysis; primary outcomes and exploratory analyses are specified below to reduce selective interpretation risk.

### Cognitive task programming

The dot-memory tasks of moderate and low difficulty were programmed using E-Prime 2.0 software, which automatically recorded participants' behavioral performance, including accuracy and reaction time, throughout the task. All participants were tested individually (one participant at a time) in a quiet room. The experimenter who conducted the ET preprocessing and statistical analyses worked with anonymized group codes and was blinded to group assignment until the primary analyses were finalized. A series of pilot studies were conducted by varying the number of moving black dots and collecting participants' subjective reports of fatigue. In the pilot work (*n* = 5), we evaluated performance (accuracy, reaction time) and subjective fatigue increase after 30 min to identify difficulty levels that were sustainable for 60 min without floor effects; tasks involving more than five dots produced near-chance responding or frequent guessing strategies in most participants within 30 min. The results indicated that tasks involving more than five dots were difficult for participants to sustain for over 30 min, often leading them to rely on guessing strategies rather than genuine cognitive processing. To ensure scientific rigor, feasibility, and validity, memory tasks exceeding five dots were excluded. Accordingly, the final paradigm defined the moderate-difficulty task as the random flashing and movement of five black dots, and the low-difficulty task as the random flashing and movement of three black dots. Task display parameters were standardized across sessions: stimuli were presented on a 24-inch LCD monitor (1920 × 1080, 60 Hz) with a fixed viewing distance of 80 cm. Monitor brightness/contrast was set to 50%/50% (factory default settings), and the same room lighting arrangement was used for all participants. Because the monitor was not photometrically calibrated and absolute luminance (cd/m^2^) was not measured, these settings are reported as operational parameters only; we do not claim precise control of absolute luminance or retinal illuminance across sessions.

### Experimental procedure

All experiments were conducted between 8:30 a.m. and 11:30 a.m. in the Psychology Laboratory of the School of Physical Education at Ningbo University. The experimental time, location, experimenters, lighting, and temperature were kept consistent for all participants. Pre- and post-measures were scheduled within the same morning window for each participant to minimize diurnal variation; the interval between the end of the task and the post-test ET recording was kept constant (approximately 10 min). Participants were instructed to maintain their normal daily routines the day before the experiment, avoid high-intensity training, strictly refrain from smoking and alcohol, and ensure adequate sleep at night. Participants were also instructed to avoid caffeine for at least 12 h before testing; adherence was checked via the arrival checklist described above. Participants were fully informed about the experimental procedures, although the exact duration of the cognitive tasks was not disclosed. They completed informed consent forms and a basic demographic questionnaire and were reminded to use the restroom, remove watches, and turn off mobile phones and other communication devices. Once all preparations were complete, participants were guided to the experimental setup and seated comfortably. Chair adjustments were made according to each participant's height to ensure a viewing distance of 80 cm from the screen, with a visual angle of 0.3 ° vertically by 0.7 ° horizontally. The experimenter provided a detailed explanation of the point memory task procedure and ET test requirements, followed by a 3-min practice session to ensure full understanding before the formal experiment began.

#### Moderate-difficulty memory task

**Figure 2:**
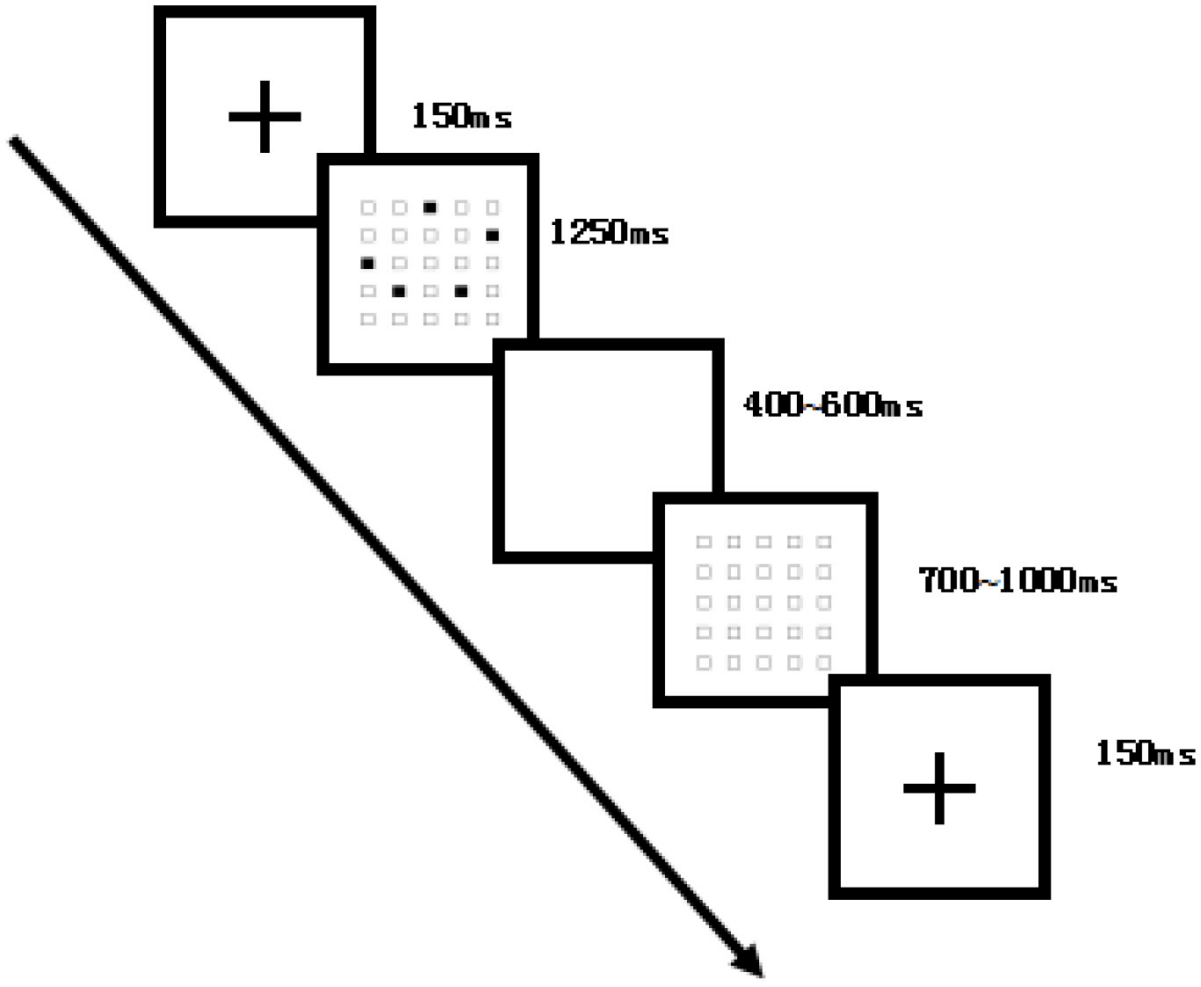
Flowchart of the dot-memory operation task (medium-difficulty example). Each trial began with a fixation cross (150 ms), followed by a 5 × 5 grid with five randomly located black dots (1,250 ms), a blank interval (400–600 ms), and then a blank 5 × 5 grid (700–1,000 ms) until response. Participants reproduced the memorized sequence by clicking on the grid. The experiment comprised four blocks of 75 trials each, for a total of 300 trials.

Following the instructions, participants pressed the spacebar to initiate a fixation cross (“+”) displayed for 150 ms. After the fixation cross disappeared, a 5 × 5 grid containing five black dots was presented for 1,250 ms, followed by an inter-stimulus interval of 400–600 ms. A blank 5 × 5 grid then appeared for 700–1,000 ms until the participant completed the memory response. Participants were required to accurately remember the sequence and positions of the dots and reproduce them by clicking on the corresponding squares with the mouse. Dot positions were randomized. The entire task consisted of four consecutive 15-min blocks, with 75 trials per block, totaling 300 trials. Behavioral indicators, including accuracy and reaction time, were recorded throughout the experiment, and average values were calculated for each block. The detailed procedure of the moderate-difficulty memory task is presented in Figure 2.

#### Low-difficulty memory task

The procedure was identical to group 1, except that only three black dots were presented in the 5 × 5 grid. Each participant completed four consecutive 15-min blocks, with 100 trials per block, totaling 400 trials. Behavioral indicators were recorded throughout and averaged for each block. The trial count differed between the two task conditions because the low-difficulty condition had shorter average response times during pilot testing, allowing more trials to fit within the fixed 15-min block duration. Because trial number may influence fatigue independently of task difficulty, this trial-count difference is treated as a potential confound and an unresolved limitation when interpreting between-task comparisons.

During the experiment, participants were instructed to remain calm and focused, minimizing eye blinks and avoiding distractions. The task was performed independently without interference, while the experimenter monitored the session in real time to handle any unexpected issues. After the experiment, participants verbally reported changes in subjective fatigue experienced during the task.

Control group procedure was standardized to reduce heterogeneity: participants remained seated in the same room for 60 min, stayed awake, and were not allowed to use phones or engage in cognitively demanding activities; permitted activities were limited to quiet sitting and viewing neutral non-task screens (e.g., a static fixation or neutral images) under supervision.

### Experimental equipment

**Figure 3:**
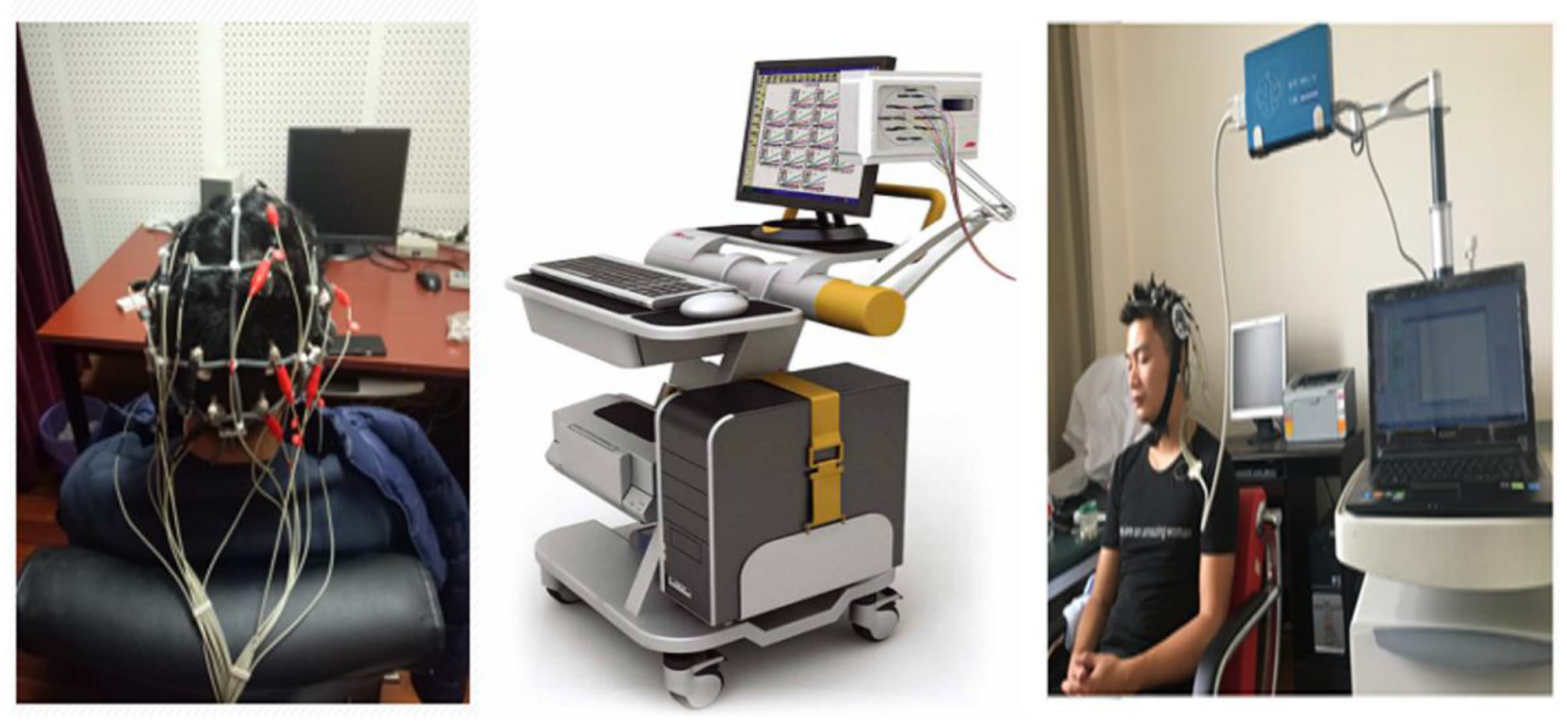
Experimental setup of the TRBX-12 (ET test) system. ET test using the TRBX-12 Motion Training State Monitoring System (Beijing Tongren Photoelectric Technology Co., Ltd.). Electrodes were placed according to the international 10–20 system with 12 unipolar leads, linked earlobes as reference, and forehead grounding. Each test lasted 18 min with participants sitting quietly, eyes closed, in an alert state.

For the ET test, electrophysiological recordings were performed using the TRBX-12 Exercise Training State Monitoring System (Beijing Tongren Photoelectric Technology Co., Ltd., China; see Figure 3). Electrodes were positioned according to the international 10–20 system, with 12 unipolar leads placed at F3, F4, C3, C4, P3, P4, O1, O2, F7, F8, T5, and T6. The linked earlobes served as reference electrodes, and a ground electrode was placed at the midline of the forehead. Prior to testing, participants remained seated quietly for approximately 10 min. The scalp and earlobes were cleaned with alcohol swabs, after which EEG electrodes were affixed at the designated sites to ensure stable contact and reliable grounding. Following calibration and stabilization of the signals, the actual recording began. Each session lasted 18 min, during which participants were instructed to keep their eyes closed, remain relaxed, stay awake, and avoid unnecessary movements. Electrode-skin impedance was kept below 5 kΩ prior to recording. Signal quality was checked for each session during preprocessing to ensure that the recording met the predefined artifact criterion, following the manufacturer's recommended guidance for TRBX-12.

### Data acquisition and indicator analysis

ET test signals were collected using the TRBX-12 Exercise Training State Monitoring System. Each recording lasted 18 min and was conducted twice: once at baseline and once immediately after the 1-h memory task. Behavioral data included accuracy and reaction time. These measures were continuously recorded throughout the 1-h memory task, divided into four 15-min intervals, and averaged within each interval for analysis.

ET preprocessing followed a predefined pipeline. Briefly, raw signals were visually inspected and automatically screened for artifacts (e.g., movement-related bursts, electrode detachment, and excessive high-amplitude transients). Segments exceeding predefined amplitude thresholds (±100 μV) or containing persistent artifacts were excluded. Channels with transient poor contact were interpolated when feasible [criteria: artifact/persistent dropout affecting < 20% of the recording and involving no more than 2 channels ( ≤ 10% of channels)]; otherwise, the recording was repeated or excluded. The final analysis required at least 80% artifact-free data to compute stable S-spectrum indices.

Neurophysiological indicators were derived from specific spectral components (S1, S2, S4, S5, S6, S7, S11, and S13), which in prior ET literature are interpreted as indices associated with neurotransmitter-system involvement: S1 for γ-aminobutyric acid (GABA), S2 for glutamic acid (Glu), S4 for 5-hydroxytryptamine (5-HT), S5 for acetylcholine (ACh), S6 for excitatory transmitters (EXC), S7 for norepinephrine (NE), S11 for dopamine (DA), and S13 for inhibitory transmitters (INH). Pre- and post-test values were compared to examine changes over time (Wu et al., 2019). For transparency, a summary table (Table 1) lists each S index, its commonly reported neurotransmitter association, and key supporting citations.

**Table 1:** Summary of S indices and commonly reported neurotransmitter associations.

**S index**	**Neurotransmitter association**	**Typical interpretation**
S1	γ-aminobutyric acid (GABA)	Primary inhibitory transmission
S2	Glutamic acid (Glu)	Primary excitatory transmission
S4	5-hydroxytryptamine (5-HT)	Serotonergic neuromodulation
S5	Acetylcholine (ACh)	Cholinergic neuromodulation (attention/memory)
S6	Excitatory transmitters (EXC)	Overall excitatory tone/E component
S7	Norepinephrine (NE)	LC–NE arousal/attention modulation
S11	Dopamine (DA)	Reward learning/motivation (RPE)
S13	Inhibitory transmitters (INH)	Overall inhibitory tone/I component

### Statistical analysis

All analyses were performed in SPSS 20.0 (IBM, USA) using two-tailed tests (α = 0.05). The primary unit of analysis was the participant. For behavioral outcomes (accuracy, reaction time), RPE, and ET-derived S indices, we used a 3 (Group: moderate-load, low-load, control) × 2 (Time: pre, post) mixed-design repeated-measures ANOVA, with Group as a between-subject factor and Time as a within-subject factor. Assumptions were evaluated via residual diagnostics (Q–Q plots/Shapiro–Wilk) and Levene's tests. Sphericity was not applicable for the Time factor because it had two levels.

Block (time-on-task) analyses. For within-task changes across the four 15-min blocks, we conducted a one-way repeated-measures ANOVA within each task cohort (moderate-difficulty and low-difficulty). For each participant, accuracy and reaction time were averaged within each block (one value per block per participant). The within-subject factor was Block (4 levels), and the error term was Participant × Block. Where *post hoc* comparisons were required, estimated marginal means (EMMs) pairwise comparisons were performed using Sidak adjustment within each outcome (accuracy or reaction time).

Multiplicity control. For ET outcomes involving multiple S indices, we controlled multiplicity using the Benjamini–Hochberg false discovery rate (BH-FDR) within each neurotransmitter-family set of indices and within each ANOVA effect family (Time, Group, and Time × Group). Pearson correlations were used to assess associations among ET indices, behavior, and RPE; within each cohort, correlation *p* values were BH-FDR-adjusted across the set of correlations tested. Effect sizes are reported as partial eta squared (η*p*^2^). All F, df, and *p* values were checked for internal consistency across text, tables, and figures. Future confirmatory studies will consider linear mixed-effects models to better address missingness and individual variability.

## Results

### Behavioral data analysis of memory tasks with different difficulty levels

#### Moderate-difficulty memory task

**Figure 4:**
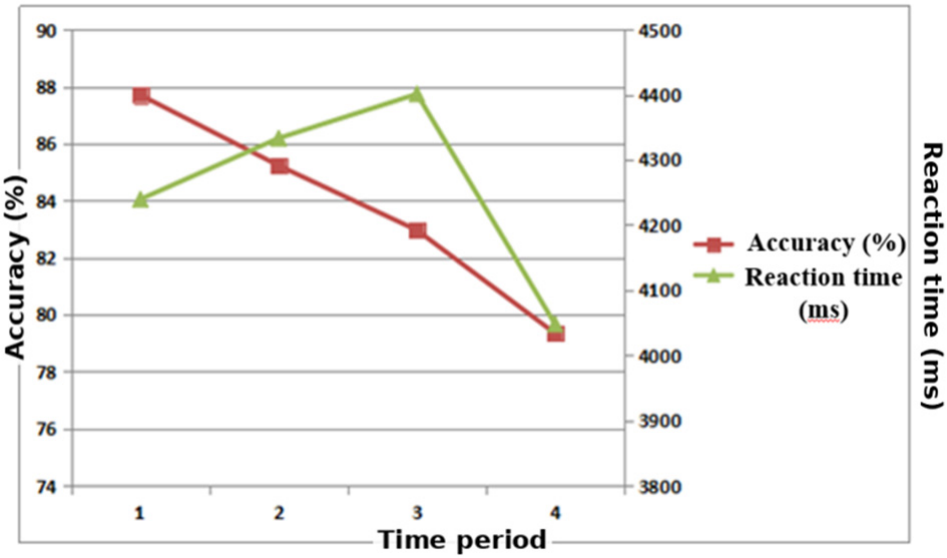
Trends in accuracy and reaction time across different time periods in a moderate-difficulty memory task. Accuracy declined progressively, with a marked drop in the fourth period (*p* < 0.01). Reaction time slowed from the first to the third period, then accelerated significantly in the fourth period (*p* < 0.05).

A one-way repeated-measures ANOVA across the four 15-min blocks showed a significant block effect on both accuracy and reaction time. Accuracy differed across blocks, *F*_(3, 42)_ = 15.352, *p* < 0.001, η*p*^2^ = 0.523. Reaction time also differed across blocks, *F*_(3, 42)_ = 2.922, *p* < 0.05, η*p*^2^ = 0.173. EMM pairwise comparisons with Sidak correction indicated that accuracy declined from Block 1 to Block 4, with the largest decrement observed in Block 4. Reaction time showed a biphasic pattern, increasing from Block 1 to Block 3 and then decreasing from Block 3 to Block 4. Mean reaction time by block was: Block 1 = 4,236 ± 520 ms, Block 2 = 4,330 ± 540 ms, Block 3 = 4,397 ± 560 ms, Block 4 = 4,046 ± 500 ms. The Block 1 vs. Block 4 contrast remained significant after correction (Sidak-adjusted *p* < 0.001). The trends in behavioral indices are illustrated in Figure 4. Accuracy–RT association (moderate difficulty). We tested the within-condition association between block-level accuracy and reaction time using participant-level block means and found a significant positive correlation (*r* = 0.55, *p* = 0.03), supporting a speed–accuracy trade-off pattern in the late blocks.

#### Low-difficulty memory task

A one-way repeated-measures ANOVA across the four 15-min blocks showed no evidence of time-on-task changes. Accuracy did not differ across blocks, *F*_(3, 42)_ = 0.266, *p* = 0.849, η*p*^2^ = 0.019, and reaction time also showed no block effect, *F*_(3, 42)_ = 0.018, *p* = 0.997, η*p*^2^ = 0.001.

### Analysis of RPE data

A 3 (Group) × 2 (Time) mixed-design repeated-measures ANOVA was performed on RPE (Table 2).

**Table 2:** Descriptive statistics of subjective fatigue at different time points (*n* = 45).

**Time point**	**Experimental group 1 (*n* = 15)**	**Experimental group 2 (*n* = 15)**	**Control group (*n* = 15)**
Time 1 (0 min)	10.68 ± 2.66	10.31 ± 2.29	10.07 ± 1.53
Time 2 (60 min)	15.69 ± 3.56	11.81 ± 1.86	11.36 ± 1.37

Data are presented as mean ± standard deviation (*n* = 45).

Experimental group 1, moderate-difficulty memory task; Experimental group 2, low-difficulty memory task; Time 1, before the experiment of memory tasks of different difficulty levels; Time 2, after 1 h of memory task experiment with different difficulty levels.

**Figure 5:**
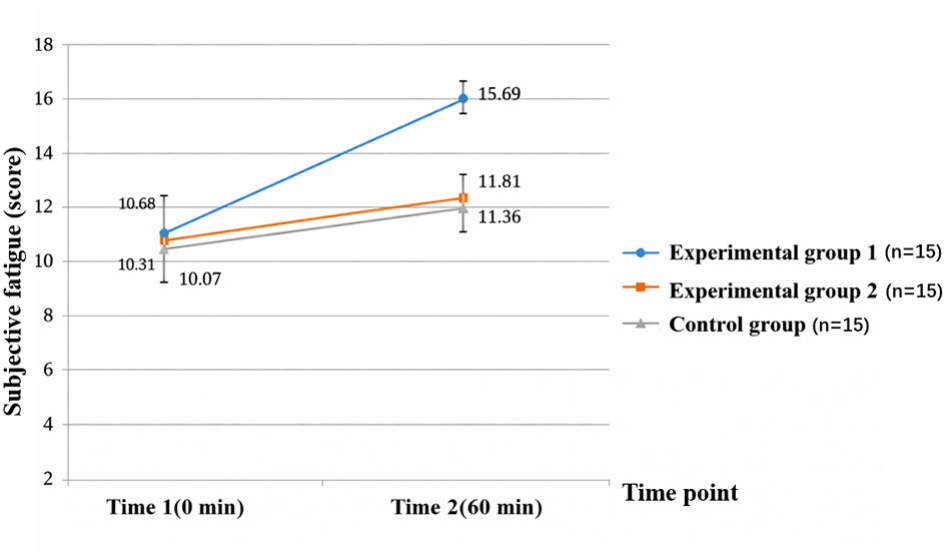
Changes in subjective fatigue pre- and post-testing in experimental group 1, experimental group 2, and control group. Experimental group 1 showed a significant increase in subjective fatigue post-experiment, while experimental group 2 and the control group showed minimal changes.

The analysis showed significant main effects of Time [*F*_(1, 42)_ = 26.537, *p* < 0.001, η*p*^2^ = 0.387] and Group [*F*_(2, 42)_ = 23.687, *p* < 0.001, η*p*^2^ = 0.530], as well as a significant Time × Group interaction [*F*_(2, 42)_ = 25.780, *p* < 0.001, η*p*^2^ = 0.551]. Simple-effects comparisons (EMMs with Sidak correction) indicated no group differences at baseline (Time 1). After the task (Time 2), RPE was highest in experimental group 1, intermediate in experimental group 2, and lowest in the control group (all Sidak-adjusted *p* < 0.01). Within-group pre–post changes were consistent with Table 2: experimental group 1 increased from 10.68 ± 2.66 to 15.69 ± 3.56 (Δ = +5.01, 95% CI = [3.60, 6.42]), experimental group 2 increased from 10.31 ± 2.29 to 11.81 ± 1.86 (Δ = +1.50, 95% CI = [0.33, 2.67]), and the control group increased from 10.07 ± 1.53 to 11.36 ± 1.37 (Δ = +1.29, 95% CI = [0.34, 2.24]). These results indicate that the moderate-difficulty memory task induced the strongest subjective fatigue response (Figure 5).

### ET indicators analysis of memory tasks with different difficulty levels

#### ET-derived indices commonly associated with inhibitory neurotransmitter systems

A 3 (Group) × 2 (Time) mixed-design repeated-measures ANOVA was conducted for inhibitory-related S indices (Tables 3, 4).

**Table 3:** Descriptive statistics of ET-derived indices commonly associated with inhibitory neurotransmitter systems.

**Index**	**Group**	**Time 1 (0 min)**	**Time 2 (60 min)**
S1	Experimental group 1	7.30 ± 3.69	3.40 ± 3.05
	Experimental group 2	5.71 ± 3.86	6.86 ± 3.58
	Control group	8.68 ± 4.24	7.37 ± 5.08
S4	Experimental group 1	19.40 ± 3.98	26.70 ± 5.53
	Experimental group 2	19.71 ± 3.86	21.32 ± 1.72
	Control group	20.26 ± 3.11	20.43 ± 2.87
S13	Experimental group 1	5.90 ± 4.67	4.60 ± 3.58
	Experimental group 2	6.29 ± 6.16	3.43 ± 1.99
	Control group	7.00 ± 3.67	7.46 ± 4.25

Data are presented as mean ± standard deviation (*n* = 45).

S1, S4, and S13 are ET-derived indices previously reported in the ET literature to be associated with inhibitory neurotransmitter-system involvement (S1, GABAergic involvement; S4, serotonergic involvement; S13, overall inhibitory tone). Experimental group 1, moderate-difficulty memory task; Experimental group 2, low-difficulty memory task; Time 1, before the experiment; Time 2, after 1 h of memory task.

**Table 4:** Results of repeated measures ANOVA for ET-derived indices commonly associated with inhibitory neurotransmitter systems.

**Index**	**Statistic**	**Within-group factor**		**Between-group factor**
		**T**	T^*^G	**G**
S1	*df*	1.42	2.42	2.42
	F	0.194	0.691	0.281
	*p*	0.662	0.507	0.757
	η*p*^2^	0.005	0.033	0.014
S4	*df*	1.42	2.42	2.42
	F	9.040	5.776	5.581
	*p*	0.004^**^	0.006^**^	0.007^**^
	η*p*^2^	0.177	0.216	0.210
S13	*df*	1.42	2.42	2.42
	F	0.893	0.111	0.695
	*p*	0.350	0.895	0.505
	η*p*^2^	0.022	0.006	0.034

T represents test time (0 min, 60 min); G represents task difficulty (moderate, low, no cognitive task); S1, S4, and S13 are ET-derived indices that have been reported in prior ET literature to be associated with inhibitory neurotransmitter-system involvement (e.g., S1 with GABAergic involvement, S4 with serotonergic involvement, and S13 with overall inhibitory tone). ^**^indicates significant effects for S4 (*p* < 0.01) for within-group and between-group factors, and a significant time × difficulty interaction (*p* < 0.01). These indices are indirect, model-derived proxies and should not be interpreted as direct neurotransmitter concentrations.

**Figure 6:**
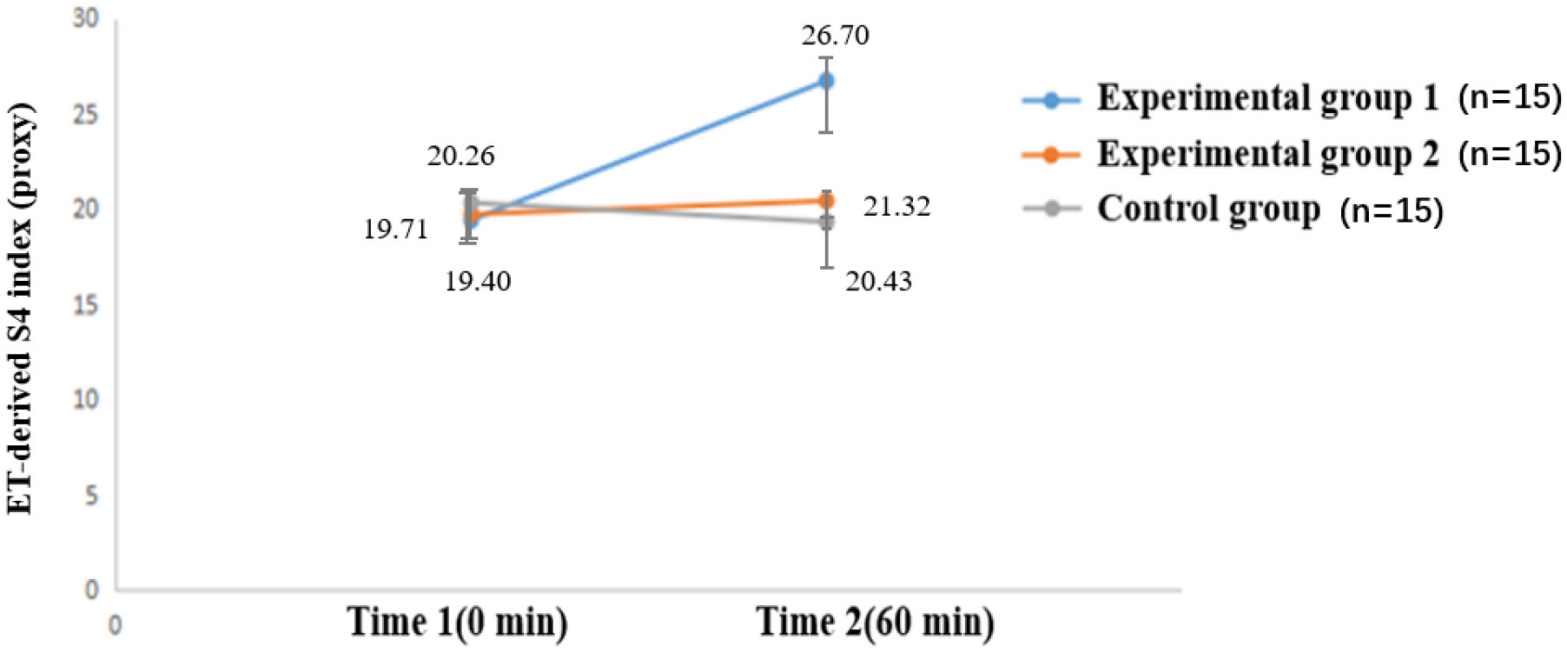
Effects of 1-h memory tasks of varying difficulty on the ET-derived S4 index (serotonergic-associated proxy in the ET literature). At Time 1 (0 min), S4 index did not differ significantly among experimental group 1, experimental group 2, and the control group (*p*>0.05). At Time 2 (60 min), S4 index were significantly higher in experimental group 1 than in experimental group 2, and higher in experimental group 2 than in the control group (*p* < 0.01). No significant changes were observed across time points in the control group or experimental group 2, whereas experimental group 1 showed a significant increase from Time 1 to Time 2 (*p* < 0.01), indicating a meaningful effect of the cognitive intervention.

For S1 and S13, neither the main effects (Time, Group) nor the interaction (Time × Group) were significant (all *p* > 0.05).In contrast, S4 showed significant effects: Time *F*_(1, 42)_ = 9.040, *p* = 0.004, η*p*^2^ = 0.177; Group *F*_(2, 42)_ = 5.581, *p* = 0.007, η*p*^2^ = 0.210; and Time × Group *F*_(2, 42)_ = 5.776, *p* = 0.006, η*p*^2^ = 0.216 (Figure 6).

Using BH-FDR correction across inhibitory indices (S1, S4, S13) within each effect family, the S4 effects remained significant: Time *q* = 0.012, Time × Group *q* = 0.018, Group *q* = 0.021.

Given the significant interaction, simple-effects comparisons (EMMs with Sidak correction) showed that at Time 1 (0 min) there were no group differences (*p* > 0.05). At Time 2 (60 min), S4 was higher in experimental group 1 than experimental group 2, and higher in experimental group 2 than the control group (*p* < 0.01). From Table 3, S4 increased in experimental group 1 (Δ = +7.30, 95% CI [4.56, 10.04]), showed a small change in experimental group 2 (Δ = +1.61, 95% CI [−0.24, 3.46]), and remained essentially stable in the control group (Δ = +0.17, 95% CI [−1.49, 1.83]).

#### ET-derived indices commonly associated with excitatory neurotransmitter systems

A 3 (Group) × 2 (Time) mixed-design repeated-measures ANOVA was conducted for excitatory-related S indices (Tables 5, 6).

**Table 5:** ET-derived indices commonly associated with excitatory neurotransmitter systems.

**Index**	**Group**	**Time 1 (0 min)**	**Time 2 (60 min)**
S2	Experimental group 1	6.65 ± 4.18	2.30 ± 2.52
	Experimental group 2	3.43 ± 4.61	6.43 ± 3.78
	Control group	5.11 ± 4.45	5.26 ± 4.08
S5	Experimental group 1	16.35 ± 4.96	17.80 ± 4.73
	Experimental group 2	15.43 ± 3.55	16.29 ± 3.38
	Control group	16.42 ± 7.00	15.05 ± 6.29
S6	Experimental group 1	14.60 ± 5.46	11.50 ± 5.55
	Experimental group 2	18.00 ± 5.16	14.14 ± 4.45
	Control group	15.47 ± 5.73	12.89 ± 6.39
S7	Experimental group 1	10.10 ± 5.70	11.00 ± 4.78
	Experimental group 2	12.14 ± 5.11	9.57 ± 4.08
	Control group	11.32 ± 7.65	10.21 ± 6.07
S11	Experimental group 1	11.00 ± 3.83	8.00 ± 3.42
	Experimental group 2	10.43 ± 2.94	7.43 ± 2.51
	Control group	10.56 ± 3.45	7.25 ± 3.54

Data are presented as mean ± standard deviation (n = 45). S2, S5, S6, S7, and S11 are ET-derived indices that have been reported in prior ET literature to be associated with excitatory/modulatory neurotransmitter-system involvement (S2, glutamatergic; S5, cholinergic; S6, overall excitatory tone; S7, noradrenergic; S11, dopaminergic). Experimental group 1, moderate-difficulty memory task; Experimental group 2, low-difficulty memory task; Time 1, before the experiment; Time 2, after 1 h of memory task.

**Table 6:** ET-derived indices commonly associated with modulatory neurotransmitter systems.

**Index**	**Statistic**	**Within-group factor**		**Between-group factor**
		**T**	T^*^G	**G**
S2	*df*	1.42	2.42	2.42
	F	0.628	2.515	0.096
	*p*	0.433	0.094	0.908
	η*p*^2^	0.015	0.112	0.005
S5	*df*	1.42	2.42	2.42
	F	4.049	0.850	1.726
	*p*	0.051	0.435	0.190
	η*p*^2^	0.094	0.041	0.079
S6	*df*	1.42	2.42	2.42
	F	0.958	1.256	1.161
	*p*	0.333	0.296	0.324
	η*p*^2^	0.023	0.059	0.055
S7	*df*	1.42	2.42	2.42
	F	0.607	2.472	2.166
	*p*	0.441	0.097	0.128
	η*p*^2^	0.015	0.110	0.098
S11	*Df*	1.42	2.42	2.42
	F	4.49	0.112	0.800
	*P*	0.040^*^	0.894	0.456
	η*p*^2^	0.092	0.006	0.038

T represents test time (0 min, 60 min); G represents task difficulty (moderate, low, no cognitive task); S2, S5, S6, S7, and S11 are ET-derived indices previously reported in the ET literature to be associated with excitatory/modulatory neurotransmitter-system involvement (indirect proxies, not direct neurotransmitter measurements). ^*^ indicates a significant main effect of time for S11 (*p* < 0.05).

For S2, S6, and S7, neither Time nor Group nor the Time × Group interaction was significant (all *p* > 0.05). For the S11, a nominal main effect of Time was observed, *F*_(1, 42)_ = 4.490, *p* = 0.040, η*p*^2^ = 0.097, while the main effect of Group and the Time × Group interaction were not significant. However, after applying BH–FDR correction across the excitatory indices (S2, S5, S6, S7, S11) within the Time-effect family, this Time effect for S11 did not survive correction (*q* = 0.200). From Table 5, S11 decreased in experimental group 1 (Δ = −3.00, 95% CI [−5.02, −0.98]), decreased in experimental group 2 (Δ = −3.00, 95% CI [−4.52, −1.48]), and decreased in the control group (Δ = −3.31, 95% CI [−5.25, −1.37]).

### Correlation analysis of the S4 index with other indicators

Pearson correlation analyses were conducted to examine relationships between S4 and behavioral measures (accuracy, reaction time) as well as RPE following the 1-h tasks (Table 7). Within each task cohort, raw *p*-values were computed for the three correlations (ACC, RT, RPE), and BH–FDR adjustment was then applied within-cohort across these three tests. After the moderate-difficulty memory task, S4 was positively correlated with accuracy (*r* = 0.607), reaction time (*r* = 0.611), and RPE (*r* = 0.514). The corresponding raw *p*-values were 0.04, 0.03, and 0.04, and BH-FDR adjustment yielded *q* = 0.04 for all three correlations. In the low-difficulty cohort, correlations were not significant (*r* = 0.312, 0.364, 0.286), with raw *p*-values of 0.15, 0.19, and 0.10 and BH-FDR-adjusted *q* = 0.19 for all three tests. These findings indicate that higher S4 index values were associated with greater task performance and subjective fatigue in the moderate-difficulty cohort, suggesting that the S4 index may be a candidate ET-derived correlate of task load and fatigue in this pilot sample.

**Table 7:** Correlation of S4 index with behavioral measures and RPE following memory tasks of varying difficulty.

**Measure**	**ACC**	**RT**	**RPE**
**S4 (Low-difficulty memory task)**			
*r*	0.312	0.364	0.286
*p* (raw)	0.15	0.19	0.10
*q* (BH–FDR)	0.19	0.19	0.19
**S4 (Moderate-difficulty memory task)**			
*r*	0.607	0.611	0.514
*p* (raw)	0.04	0.03	0.04
*q* (BH–FDR)	0.04^*^	0.04^*^	0.04^*^

ACC, accuracy; RT, reaction time; RPE, subjective fatigue. *p* values are raw (unadjusted). *q* values are BH–FDR-adjusted within each task cohort across the three correlations (ACC, RT, RPE). S4 is an ET-derived index previously proposed to reflect serotonergic-system involvement in the ET literature (indirect proxy, not a direct 5-HT measure). ^*^ indicates *q* < 0.05.

## Discussion

### Behavioral indicator trends

This study employed 1-h dot memory tasks of varying difficulty to induce central fatigue in participants. The task required participants to rapidly memorize both the number and sequence of dots presented on the screen and then reproduce them accurately within a short period, continuing until clear signs of fatigue emerged.

Behavioral results (Figure 4) showed that under moderate task difficulty, accuracy declined progressively with task duration, while RPE increased significantly, indicating a gradual intensification of subjective fatigue. This pattern is consistent with the cognitive-fatigue literature describing vigilance decrement and reduced sustained attentional control during prolonged tasks, which typically manifests as declining accuracy and rising subjective effort with time-on-task. This trend is consistent with previous findings in memory and attention tasks. For example, in figure-matching memory tasks, accuracy is typically highest at the beginning and then gradually decreases (Zhou et al., 2015). Similarly, Wu et al. (2019) reported a significant decline in accuracy with prolonged Flanker task performance, and Liu et al. (2016) obtained comparable results. In the present study, participants performing the moderate dot memory task commonly exhibited signs of fatigue over time, including inattention, mental lapses, irritability, slowed responses, and elevated RPE. These findings suggest the emergence of central fatigue accompanied by behavioral deterioration.

Reaction time showed a distinct “slower-then-faster” trajectory, with an initial prolongation followed by a shortening toward the final block. This result diverges from some earlier studies. For example, Liu et al. (2010) reported that a 60-min spatial memory task produced prolonged reaction times in later phases, with similar findings reported by Cao et al. (2003) and Liu (2004). In contrast, other studies align with our results. Liu et al. (2016), using eye-tracking technology to examine college athletes after 1 h of Flanker tasks, found that reaction times were slower at the beginning and subsequently shortened. To directly evaluate whether the late-stage acceleration reflects a behavioral speed–accuracy trade-off rather than improved processing efficiency, we examined the within-condition association between block-level accuracy and reaction time using participant-level block means. The significant positive accuracy–RT correlation (*r* = 0.55, *p* = 0.03) indicates that blocks with longer reaction times tended to show higher accuracy, whereas blocks with shorter reaction times tended to show lower accuracy, supporting a speed–accuracy trade-off pattern in the late blocks. Alternative explanations remain plausible and are not mutually exclusive. Strategy change or reduced response caution in the final block (e.g., more guessing) can shorten reaction times without reflecting improved processing efficiency. Practice effects alone are unlikely to explain the late-stage acceleration because accuracy continued to decline, but practice may still contribute early in the task. College athletes, in particular, may have lower tolerance for prolonged sedentary cognitive tasks, and as fatigue accumulates they may adopt effort-avoidance strategies. Observations and post-task interviews further revealed negative emotional states (restlessness, irritability), difficulty maintaining attention, and increased irrelevant movements in later phases. These factors likely contributed to reduced engagement, lower accuracy, and shortened reaction times.

Taken together, the findings indicate that 1 h of moderate dot memory tasks results in a continuous decline in accuracy, a “slower-then-faster” pattern in reaction time, and a significant increase in RPE, reflecting the onset of central fatigue and associated behavioral impairment (Boksem et al., 2006; Boksem and Tops, 2008). Framing these results within cognitive-fatigue models, the accuracy decline is consistent with vigilance decrement, while the reaction-time pattern is compatible with changing response caution and speed-accuracy trade-off under sustained effort. Accuracy and reaction time thus appear to be important behavioral indicators for assessing central fatigue. However, while the decline in accuracy is a consistent outcome across studies, the direction of reaction time changes remains inconclusive. Such discrepancies may be attributable to differences in task paradigms or to variations in participants' adaptability and tolerance to cognitive workload. In the low-difficulty condition, we avoid interpreting non-significant block effects as evidence of “no change,” given the pilot sample size. Instead, we emphasize effect sizes and descriptive interpretation: block effects were non-significant with very small effect sizes, suggesting that any time-on-task change, if present, is likely small under low task demand and should be tested in larger confirmatory studies with adequate power and joint modeling of accuracy and reaction time.

### ET-derived indices under different memory task difficulties

The present study examined ET-derived neurophysiological indices during a 1-h dot-memory task under different cognitive loads. Consistent with the observed data pattern, the ET-derived S4 index showed a group-dependent increase after the moderate-difficulty task compared with baseline and with the low-difficulty and control conditions. Importantly, ET does not directly quantify neurotransmitter concentrations or receptor activity; therefore, we report this finding as a change in an ET-derived S-index. In the ET literature, S4 has been proposed to reflect serotonergic-system involvement, and thus a cautious interpretation is that the S4 increase may be compatible with altered serotonergic involvement during time-on-task fatigue. This interpretation remains model-derived and hypothesis-generating, rather than confirmatory.

This S4 pattern is discussed here in the broader context of fatigue research where serotonergic processes have been reported to change alongside fatigue-related states (Blomstrand et al., 1995; Fernstrom and Fernstrom, 2006; Wu et al., 2019). However, these studies do not constitute gold-standard validation of ET indices, and they should not be taken to imply that our ET results reflect direct measurements of serotonergic activity. Instead, they provide relevant background that motivates cautious mechanistic hypotheses about how serotonergic involvement might relate to fatigue under sustained task engagement. In the present study, the intervention-related change was more pronounced under moderate difficulty at the level of the ET-derived S4 index, suggesting that task load may modulate ET-derived signatures of fatigue.

We emphasize that several physiological mechanisms often discussed in the literature were not directly measured in this experiment. For example, one speculative explanation is that prolonged engagement in demanding memory operations could increase metabolic demand and potentially contribute to energetic stress, which might in turn influence systems implicated in fatigue regulation. Some studies have linked fatigue-related states to changes in serotonergic markers and associated metabolic conditions (Meeusen et al., 2007; Gharakhanlou and Fasihi, 2023). In addition, hypotheses involving altered synthesis and degradation dynamics of serotonin have been proposed in related contexts (Martínez-Díaz et al., 2020; Nourpetelian, 2022). Nevertheless, we did not measure blood glucose, oxygenation, ATP, or enzymatic activity, and we did not directly quantify serotonin concentration. Therefore, any references to metabolic stress, ATP depletion, enzyme activity, or accelerated 5-HT synthesis should be understood as tentative and indirect explanatory possibilities that require dedicated validation with independent physiological measures, rather than as conclusions supported by the present ET data.

In contrast, we did not observe significant task-specific changes in other ET-derived indices commonly discussed in relation to inhibitory neurotransmitter systems (e.g., indices previously linked to GABAergic involvement). This discrepancy from some earlier findings (Kalueff and Nutt, 2007; Gu, 2021) may reflect differences in task paradigms: prior studies largely focused on high-intensity physical exercise, whereas the present study employed a cognitive task. An additional explanation is methodological sensitivity: ET indices may differ in responsiveness across neurotransmitter-associated bands over a 1-h cognitive protocol, and the pilot sample size may limit power to detect smaller effects. Overall, the present results indicate a selective change in the ET-derived S4 index under moderate cognitive load, while broader inhibitory-system conclusions remain conservative. In this study, ET-derived indices commonly discussed as excitatory or modulatory did not show robust task-specific alterations, which contrasts with some prior findings based on exercise-induced fatigue (Tornero-Aguilera et al., 2022; Naoi et al., 2025). One explanation is that cognitive tasks, such as the 1-h memory operation used here, may induce a different fatigue profile and may be less intense than physically demanding exercise protocols, limiting direct comparability. Notably, the S11 index (discussed as DA-associated in some ET reports) showed a main effect of time but no group difference. This suggests a non-specific time-related shift that may reflect general factors shared across conditions, such as prolonged sitting, habituation to the laboratory environment, expectation effects, or measurement drift, rather than task-load–specific cognitive fatigue. This pattern is also compatible with the possibility that dopaminergic involvement differs between cognitive-task–induced fatigue and exercise-induced fatigue, where motivational and motor activation components can be more prominent. These interpretations should be tested by designs that include stronger manipulation checks, longer protocols, or combined cognitive and physical loading.

Although the present results did not reveal meaningful task-specific changes in ET-derived indices commonly linked to excitatory systems, it is premature to conclude that 1-h cognitive tasks have no effect. Future studies could increase sensitivity by extending task duration, increasing difficulty, or using dual-demand paradigms (cognitive plus physical) to better approximate sport contexts, while also increasing sample size to improve power for interaction effects. This experiment was conducted under strictly controlled laboratory conditions using a dot-memory task paradigm. Although this differs from memory-related tasks embedded in sports training and exercise contexts, the essential process remains short-term information processing in the brain. From the perspective of central information processing, including encoding, filtering, storage, retrieval, judgment, decision-making, and behavioral responses, these tasks are fundamentally similar despite differences in symbol type, information load, intensity, or tempo. Therefore, long-duration cognitive processing tasks, whether performed in front of a computer or during complex motor training, may induce central fatigue through mechanisms that could involve multiple systems. The behavioral manifestations observed in this study—decreased accuracy and altered reaction times—may also occur in athletes during prolonged training or competition, as well as in general exercise settings. Accordingly, our findings may provide valuable insights for explaining cognitive fatigue in sports contexts. In summary, prolonged memory operations can induce central fatigue characterized by behavioral changes and may be accompanied by changes in ET-derived indices (including the S4 index). At the same time, conclusions should remain proportional to the evidence: the study supports a moderate-load task effect on behavioral fatigue markers and on the ET-derived S4 index, whereas mechanistic explanations and broader neurotransmitter interpretations remain tentative and should be evaluated in confirmatory designs. These results hold important implications for the evaluation and optimization of training programs and fitness activities, and for preventing central fatigue in both athletic and general populations.

### Strengths and limitations

This study has several limitations. First, the reaction-time pattern observed here was not fully consistent with some previous reports, which may reflect the specific characteristics of the sample and the modest sample size. The participants were university athletes and may not represent athletes from different sport disciplines, training volumes, or performance levels, nor the general population. Individual differences in cognitive ability, personality, and behavioral habits may also influence task strategy and adaptability (Eysenck, 1957). Second, ET provides indirect, model-derived indices that are interpreted as reflecting neurotransmitter-system involvement, rather than direct measurements of neurotransmitter concentration or receptor activity. The biophysical basis and external validation of ET remain debated, which constitutes a major limitation when drawing mechanistic conclusions from S indices. Consequently, conclusions regarding neurotransmitter mechanisms should be treated as tentative and require independent verification. In addition, due to resource constraints, we did not include other physiological measures, which restricts multi-modal triangulation of fatigue mechanisms. Future studies should combine ET with established behavioral and neurophysiological markers of cognitive fatigue (e.g., vigilance-related outcomes, eye-tracking indices, and conventional EEG/ERP measures), and where feasible, further validate ET indices using pharmacological approaches or neuroimaging techniques such as PET. Third, the present protocol used a 1-h dot-memory task in a controlled laboratory setting. Whether longer durations, higher difficulty, or combined cognitive–physical loading would elicit broader neurophysiological changes remains unknown. The cross-sectional, single-session design also limits inference about long-term adaptation, cumulative training effects, or chronic fatigue states; longitudinal designs across training cycles are needed. In addition, the two task conditions differed in total trial counts (300 vs. 400) due to response-time differences under the fixed block duration. Because trial number may contribute to fatigue independently of difficulty, this difference represents a potential confound that cannot be fully disentangled in the current design and should be addressed in future studies by equating trial counts or total response demands across conditions. Finally, while the paradigm was well-controlled, its ecological validity is limited relative to sport-specific decision-making under fatigue. Future work should examine whether similar ET patterns emerge in more realistic tasks embedded in sport-specific drills or competition-like conditions, and should extend the protocol to non-athlete and clinical populations to evaluate generalizability and translational value.

Despite these limitations, this study demonstrates several important strengths. It provides a controlled test of cognitive-task–induced fatigue using a pre–post mixed design and integrates behavioral performance, subjective fatigue (RPE), and ET-derived indices within the same protocol. The results suggest that moderate cognitive load produces clear behavioral fatigue markers and a selective increase in the ET-derived S4 index after the task compared with baseline and other groups, consistent with subjective fatigue changes. The dissociation between accuracy and reaction time also highlights the importance of considering speed–accuracy trade-offs and strategy shifts when interpreting behavioral fatigue. Finally, the differential responsiveness across ET indices generates specific, testable hypotheses for future confirmatory studies with improved validation and greater statistical power.

## Conclusion

This study demonstrates that a 1-h moderate-difficulty memory task effectively induces central fatigue. Behaviorally, task accuracy declined over time and reaction times showed initial slowing followed by acceleration, consistent with time-on-task effects and potential speed–accuracy trade-offs. Neurophysiologically, the moderate-difficulty task was accompanied by an increase in the ET-derived S4 index, whereas other ET-derived indices showed no consistent task-specific changes. Because ET indices are indirect, model-derived proxies, the neurochemical interpretation of S4 (serotonergic involvement) should be viewed as tentative and hypothesis-generating. These findings provide a behavioral and ET-based profile of cognitive fatigue during prolonged memory tasks in athletes and motivate future multi-modal validation studies.

## Practical applications

The present findings indicate that a 1-h moderate-load memory task can induce measurable cognitive fatigue, reflected in deteriorating behavioral performance and increased subjective fatigue, alongside a selective change in the ET-derived S4 index. In applied sport settings, this supports the view that cognitive load, in addition to physical load, should be monitored during training periods that involve intensive tactical learning, video analysis, or prolonged concentration demands.

Practically, simple behavioral indicators (accuracy and reaction time) combined with RPE can be used as low-cost markers to track cognitive state and to guide adjustments in session structure, including the timing of cognitively demanding drills, the allocation of recovery intervals, and the distribution of technical vs. tactical learning. Where ET is available, ET-derived indices may provide an additional physiological layer for monitoring fatigue-related changes, but results should be interpreted as indirect markers rather than direct neurotransmitter measurements. Finally, the observed pattern of fatigue suggests that interventions aiming to reduce excessive cognitive load or improve recovery (e.g., structured rest breaks, sleep and recovery management, relaxation strategies, and individualized workload planning) may help mitigate the impact of cognitive fatigue on training quality and performance.

## Data Availability

The original contributions presented in the study are included in the article/supplementary material, further inquiries can be directed to the corresponding authors.
